# The White-Rot Basidiomycete *Dichomitus squalens* Shows Highly Specific Transcriptional Response to Lignocellulose-Related Aromatic Compounds

**DOI:** 10.3389/fbioe.2019.00229

**Published:** 2019-09-20

**Authors:** Joanna E. Kowalczyk, Mao Peng, Megan Pawlowski, Anna Lipzen, Vivian Ng, Vasanth Singan, Mei Wang, Igor V. Grigoriev, Miia R. Mäkelä

**Affiliations:** ^1^Department of Microbiology, University of Helsinki, Helsinki, Finland; ^2^Fungal Physiology, Westerdijk Fungal Biodiversity Institute and Fungal Molecular Physiology, Utrecht University, Utrecht, Netherlands; ^3^U.S. Department of Energy Joint Genome Institute, Walnut Creek, CA, United States

**Keywords:** transcriptome, gene expression, basidiomycete, *Dichomitus squalens*, aromatic compounds, lignocellulose, lignin, platform chemicals

## Abstract

Lignocellulosic plant biomass is an important feedstock for bio-based economy. In particular, it is an abundant renewable source of aromatic compounds, which are present as part of lignin, as side-groups of xylan and pectin, and in other forms, such as tannins. As filamentous fungi are the main organisms that modify and degrade lignocellulose, they have developed a versatile metabolism to convert the aromatic compounds that are toxic at relatively low concentrations to less toxic ones. During this process, fungi form metabolites some of which represent high-value platform chemicals or important chemical building blocks, such as benzoic, vanillic, and protocatechuic acid. Especially basidiomycete white-rot fungi with unique ability to degrade the recalcitrant lignin polymer are expected to perform highly efficient enzymatic conversions of aromatic compounds, thus having huge potential for biotechnological exploitation. However, the aromatic metabolism of basidiomycete fungi is poorly studied and knowledge on them is based on the combined results of studies in variety of species, leaving the overall picture in each organism unclear. *Dichomitus squalens* is an efficiently wood-degrading white-rot basidiomycete that produces a diverse set of extracellular enzymes targeted for lignocellulose degradation, including oxidative enzymes that act on lignin. Our recent study showed that several intra- and extracellular aromatic compounds were produced when *D. squalens* was cultivated on spruce wood, indicating also versatile aromatic metabolic abilities for this species. In order to provide the first molecular level systematic insight into the conversion of plant biomass derived aromatic compounds by basidiomycete fungi, we analyzed the transcriptomes of *D. squalens* when grown with 10 different lignocellulose-related aromatic monomers. Significant differences for example with respect to the expression of lignocellulose degradation related genes, but also putative genes encoding transporters and catabolic pathway genes were observed between the cultivations supplemented with the different aromatic compounds. The results demonstrate that the transcriptional response of *D. squalens* is highly dependent on the specific aromatic compounds present suggesting that instead of a common regulatory system, fine-tuned regulation is needed for aromatic metabolism.

## Introduction

Non-edible lignocellulosic biomass is increasingly researched as a sustainable alternative to fossil fuel-based energy sources, biomaterials, and chemicals. Majority of lignocellulose waste originates from forestry (e.g., bark, logging debris, and sawdust) or agriculture (e.g., rice and wheat straw, corn stover and sugar cane bagasse) and is mainly composed of cellulose, hemicelluloses, and lignin. Lignin is an aromatic polymer and the most recalcitrant constituent present in woody plant cell walls, where it e.g., provides rigidity, resistance against microbial invasion and facilitates water transportation (Tolbert et al., 2014). In softwoods, the amount of lignin can be up to 32% of plant dry weight (Sjöström, 1993), and it is the most abundant renewable source of aromatic compounds on earth. Lower amounts of aromatic compounds are also present in plant biomass as side-groups of polysaccharides xylan and pectin, as well as in tannins (McLeod, 1974; Mäkelä et al., 2015). Aromatics derived from plant biomass have applications in various industrial sectors, e.g., as precursors for synthesis of biopolymers (Kawaguchi et al., 2017; Feghali et al., 2018; Kohlstedt et al., 2018). Therefore, lignocellulose holds a great potential as a source of chemical building blocks for the sustainable production of valuable compounds in biorefineries.

Despite huge prospects, lignin remains the least utilized polymer in lignocellulose (Rinaldi et al., 2016). Degradation of lignin is difficult due to its insolubility and complex, random structure with various non-hydrolysable intramolecular C-C, C-O, and β-aryl ether bonds (Hatakka and Hammel, 2011). Therefore, the vast majority of lignin, which is formed as major byproduct of the wood-related biorefineries as well as pulp and paper industry, is currently being used for low-value production of heat and electricity (Calvo-Flores and Dobado, 2010). However, fragmentation of lignin can be achieved by physical and/or chemical methods, and several phenolic compounds, such as *p*-coumaric acid, *p*-hydroxybenzoic acid, ferulic acid, vanillin, and vanillic acid, are already produced from lignin via chemical oxidation or pyrolysis (Otto and Simpson, 2006; Li et al., 2015). Nowadays, with the global move toward the bio-based economy, great attention is given to development of environmentally friendly modification methods of lignocellulose, such as enzymatic conversion (Den et al., 2018).

Basidiomycete white-rot fungi are the only organisms that are able to degrade all polymers present in lignocellulose, including high molecular weight native lignin molecules (Hatakka and Hammel, 2011). During this process, fungi form metabolites some of which represent high-value platform chemicals or industrially important chemical building blocks, such as benzoic and protocatechuic acid (Lubbers et al., 2019). While, the extracellular plant biomass degrading enzyme systems of the white-rot fungi have been extensively studied (Mäkelä et al., 2014; Rytioja et al., 2014; Manavalan et al., 2015; An et al., 2019), the knowledge on their metabolism converting the resulting small aromatic compounds is still far from complete. For example, instead of systematic characterization of full metabolic pathways, mainly single conversions of specific compounds have been studied (Mäkelä et al., 2015; Lubbers et al., 2019). It should also be noted that many wood-degrading basidiomycetes have been reported to synthesize aromatic compounds such as vanillin and veratryl alcohol (Harper et al., 1990; Lomascolo et al., 1999).

*Dichomitus squalens* is an efficient wood-degrading white-rot fungus that predominantly degrades softwood (Andrews and Gill, 1943; Renvall et al., 1991), but can also grow on hardwoods (Blanchette et al., 1987) in nature. *D. squalens* is a promising reference species to investigate white-rot fungal plant biomass degradation, as it has a flexible physiology to utilize different types of biomass as sources of carbon and energy (Rytioja et al., 2017; Daly et al., 2018). We recently showed that *D. squalens* produces several intra- and extracellular aromatic compounds during cultivation of on wood (Daly et al., 2018) and characterized the first functional β-*O*-4 bond cleaving fungal β-etherase (GST1) from this species (Marinović et al., 2018). All these aspects higlight the suitability of *D. squalens* for studies on fungal metabolism of lignocellulose-related aromatic compounds.

In this study, we aimed to provide the first systematic, molecular level insight into the white-rot fungal response to plant biomass related aromatic monomers. For this, we used RNA sequencing (RNA-seq) to identify all differentially expressed transcripts in *D. squalens* when the fungus was exposed to 10 different monomeric aromatic compounds in comparison with control conditions without aromatic compounds. The aromatic compounds included cinnamic acid, which in lignin biosynthesis can be converted to the three monolignol building blocks of lignin, i.e., coniferyl, sinapyl, and *p-*coumaryl alcohol (Humphreys and Chapple, 2002), coniferyl alcohol, which is one of the monolignols (Vanholme et al., 2010), and eight putative metabolic conversion products of lignin (ferulic acid, vanillin, vanillyl alcohol, vanillic acid, protocatechuic acid, veratryl alcohol, *p*-coumaric acid, *p*-hydroxybenzoic acid). Regulons for each aromatic compound were defined as the number of genes with differential expression when compared to control conditions. Significant differences were observed between the studied cultivations, i.e., with respect to the expression of genes with predicted intracellular oxidative activity including oxidoreductases, alcohol dehydrogenases, and cytochrome P450 monooxygenases, showing that the transcriptional response of *D. squalens* is highly dependent on the specific aromatic compounds present.

## Materials and Methods

### Fungal Strain and Growth Conditions

*Dichomitus squalens* dikaryotic strain FBCC312 was obtained from the FBCC-HAMBI culture collection (www.helsinki.fi/hambi/) and maintained on 2% (w/v) malt extract 2% (w/v) agar (MEA) plates. All other cultivations were inoculated with single mycelium-covered agar plug (0.5 cm in diameter) from a freshly growing MEA plate. Stocks of ferulic acid (Sigma), vanillyl alcohol (Fluka), vanillin (Merck), vanillic acid (Fluka), *p*-coumaric acid (Sigma), protocatechuic acid (Sigma), *p*-hydroxybenzoic acid (Fluka), cinnamic acid (Merck), veratryl alcohol (Fluka), or coniferyl alcohol (gift from the Department of Chemistry, University of Helsinki) were prepared by dissolving in 40% (v/v) dimethyl sulfoxide (DMSO; VWR Chemicals) to the final concentration of 40 mM. The inhibiting effect of the tested aromatics and/or DMSO on *D. squalens* was assessed by growing the fungus on low-nitrogen asparagine-succinate (LN-AS, pH 4.5) 1.5% (w/v) agar plates with 0.05% (v/v) glycerol (Hatakka and Uusi-Rauva, 1983) and supplemented with 0.2, 0.5, or 1 mM aromatic compounds, which contained 0.2, 0.5, or 1% DMSO, respectively. The duplicate plates were inoculated with a centrally placed agar plug and incubated at 28°C in the dark for 4 days. The toxicity was assessed by measuring the diameter of the radial growth of fungal colony in comparison to the plates without aromatic compounds.

For the gene expression analyses, the fungus was grown on LN-AS agar plates containing 0.05% (v/v) glycerol and 0.5% (v/v) DMSO (no aromatics control) or 0.05% (v/v) glycerol and 0.5 mM of one of the mentioned aromatic compounds as the only carbon sources. Before inoculation with an agar plug, each plate was covered with a sterile polycarbonate membrane (GVS Life Sciences) to facilitate the harvesting of the mycelia. After 4 days of growth at 28°C, mycelium from the outer ring (1.5 cm wide) of the fungal colony was carefully scraped with an RNase-free spatula and flash frozen in liquid nitrogen. For each condition, three biological replicate cultures were performed.

### Preparation and Sequencing of RNA

*Dichomitus squalens* RNA was extracted from the cultivations supplemented with the 10 different aromatic compounds and analyzed by RNA sequencing (RNA-seq). First, frozen mycelia were transferred to a pre-chilled 2 mL lysing matrix tube (MP Biomedicals) with 1 mL of TRIzol (Sigma) and ground in a tissue homogenizer (FastPrep-24™, MP Biomedicals) for 2 × 10 s at maximum speed, with a cooling on ice between the grinding. After 5 min incubation at RT, 0.2 mL chloroform was added, the tubes were shaken vigorously and incubated for additional 3 min at RT. Then, the samples were centrifuged for 10 min, 13,000 × g, at 4°C and the aqueous phase containing RNA was carefully collected and processed using the NucleoSpin RNA II purification kit (Macherey-Nagel) according to manufacturer's instructions. The quantity and integrity of RNA were measured with NanoDrop One Microvolume UV-Vis Spectrophotometer (Thermo Scientific) and RNA600 Nano Assay using the Agilent 2100 Bioanalyzer (Agilent Technologies, USA).

Purification of mRNA, synthesis of cDNA library and sequencing on the Illumina HiSeq2500 platform were performed at the Joint Genome Institute (JGI, Walnut Creek, USA) as described previously (Daly et al., 2018). One sample from veratryl alcohol cultivation did not pass the quality control and was removed from the sequencing queue.

The monokaryotic *D. squalens* CBS464.89 strain derived from the FBCC312 dikaryon (Pham et al., 1990; Casado López et al., 2017) is currently the best *D. squalens* reference genome available (Casado López et al., 2019), and was therefore used to map the filtered reads from each library. The reads from each of the RNA-seq samples were deposited in the Sequence Read Archive at NCBI with individual sample BioProject Accession numbers (PRJNA500193 to PRJNA500234).

### RNA-Seq Data Analysis

Gene expression levels were measured as Fragments Per Kilobase of transcript per Million mapped reads or FPKM (Trapnell et al., 2010). Genes with FPKM > 70 and FPKM < 10 were considered highly and lowly expressed, respectively, and genes with 10–70 FPKM moderately expressed. The correlation matrix and principal component analysis (PCA) were performed using the corrplot and FactoMineR package (Lê et al., 2008), respectively, in R version 3.5.0.

Transcript levels of the samples cultivated with glycerol and aromatic compounds (ferulic acid, vanillyl alcohol, vanillin, vanillic acid, *p*-coumaric acid, protocatechuic acid, *p*-hydroxybenzoic acid, cinnamic acid, veratryl alcohol, or coniferyl alcohol) were compared to the control cultures without aromatics using DESeq2 version 1.10.0 (Love et al., 2014). Between each pair of conditions, differentially expressed genes with fold change > 2, adjusted *p* < 0.01, and FPKM > 10 in at least one condition were identified. Functional annotation of differentially expressed genes was based on combined information from EuKaryotic Orthologous Groups (KOG), Kyoto Encyclopedia of Genes and Genomes (KEGG) pathway mapping, InterPro protein sequence analysis & classification, and Carbohydrate-Active enZymes (CAZy) classifications for *D. squalens* CBS464.89 (Dicsqu464_1) retrieved from JGI MycoCosm database (https://genome.jgi.doe.gov/cgi-bin/kogBrowser?db=Dicsqu464_1) and updated with information from Daly et al. (2018). Genes with annotation in more than one database were manually assigned into one of the four main functional groups created based on KOG system: “Carbohydrate-Active enzyme (CAZyme),” “Metabolism,” “Cellular processes and signaling,” or “Information storage and processing.” Genes lacking well-defined annotation or present in only one database were collected in “General function prediction only” and genes without any available annotation in “Not annotated” groups, respectively.

The statistically overrepresented Gene Ontology (GO) terms were analyzed using the Biological Networks Gene Ontology (BiNGO) plugin in the Cytoscape v 3.6.0 software (Maere et al., 2005), with custom input Dicsqu464_1 GO annotation retrieved from JGI MycoCosm database (https://genome.jgi.doe.gov/cgi-bin/kogBrowser?db=Dicsqu464_1). Hierarchical clustering heatmaps were made using gplots package in R, with the complete-linkage clustering method and Euclidean distance. Intersection groups, representing unique sets of gene identified only between intersected elements, were visualized using UpSetR package v1.3.3 in R. Protein sequences of previously identified bacterial and fungal aromatic metabolic genes were retrieved from National Center for Biotechnology Information (NCBI, https://www.ncbi.nlm.nih.gov/) based on information collected in Lubbers et al. (2019) and used as a query for homology search in Dicsqu464_1 genome.

## Results

### Growth of *D. squalens* on Different Aromatic Monomers

In this study, we aimed to provide the first molecular level systematic insight into the conversion of lignocellulose-related aromatic monomers by the basidiomycete fungus *D. squalens*. Ten compounds were chosen for the comparative analysis: coniferyl alcohol, ferulic acid, vanillin, vanillyl alcohol, vanillic acid, veratryl alcohol, protocatechuic acid, *p*-coumaric acid, *p*-hydroxybenzoic acid, and cinnamic acid. Cinnamic acid is a plant L-phenylalanine-derived compound that can be converted via several biosynthetic steps into three different phenylpropanoid precursors of lignin (monolignols), including coniferyl alcohol (Leisola et al., 2012; Wang et al., 2013). The remaining monomeric aromatic compounds are predicted to be intermediate products of aromatic metabolism in filamentous fungi (Mäkelä et al., 2015). Vanillin and *p*-coumaric acid were previously reported to be formed during lignin degradation by several bacterial species (Lubbers et al., 2019), and ferulic acid was chosen because it is commonly present in biomass as e.g., substituent of xylans. Additionally, *p*-hydroxybenzoic acid, protocatechuic acid and vanillic acid were identified in the metabolome of *D. squalens* grown on spruce wood for two and four weeks (Daly et al., 2018), while *p*-hydroxybenzoic acid and vanillic acid have also been formed during spruce degradation by the white-rot fungus *Phanerochaete chrysosporium* (Chen et al., 1982).

**Figure 1:**
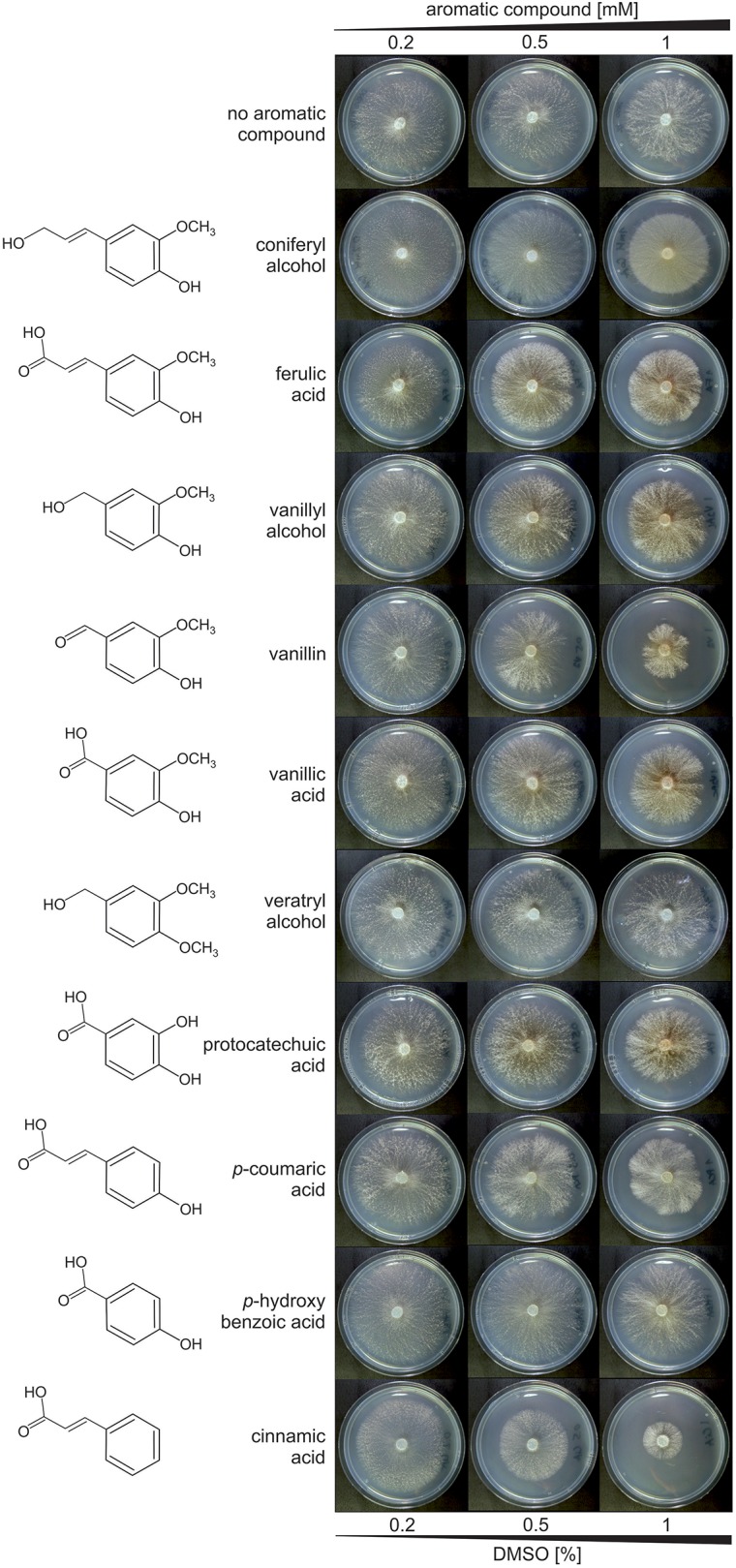
*D. squalens* FBCC312 grown on LN-AS agar medium supplemented with 0.05% glycerol and increasing concentrations (0.2, 0.5, 1 mM) of selected aromatic compounds. Several tested aromatics had inhibitory effect on fungal growth at 1 mM, and therefore the final concentration of 0.5 mM was used for transcriptome induction. Low concentrations of DMSO (0.2–0.5%) used as a solvent for the aromatic compounds did not affect fungal growth. Plates were incubated for 4 days at 28°C.

Many aromatic compounds have inhibitory effect on fungal growth even at low concentrations (Adeboye et al., 2014; Lima et al., 2018). Therefore, the influence of the selected aromatics on the growth of *D. squalens* was tested in final concentrations of 0.2, 0.5, and 1 mM. The growth medium was additionally supplemented with 0.05% glycerol due to previous reports showing that many white-rot fungi metabolize lignin-related aromatic compounds only in the presence of an alternate carbon and energy source (Kirk and Farrel, 1987). None of the tested aromatic compounds had inhibitory effect of the growth of *D. squalens* determined as the diameter of the colony at final concentration 0.2 mM, while at 0.5 mM small growth reduction was observed on vanillin and cinnamic acid (Figure 1). In final concentration of 1 mM, all aromatics, except *p*-hydroxybenzoic acid, inhibited the growth of *D. squalens*. Vanillin and cinnamic acid restricted the growth most. All aromatic compounds were dissolved in 40% DMSO, since DMSO was previously reported as an optimal solvent for Kraft lignin without affecting activity of the main lignin-degrading enzymes in the white-rot fungus *Coriolus (Trametes) versicolor* (Brzonova et al., 2017). The radial growth of *D. squalens* was not reduced by the presence of 0.2–0.5% DMSO as observed from the control cultivations without addition of aromatic compound (Figure 1). However, a small reduction in the growth of the fungal colony was observed with 1% DMSO. Based on these results, an intermediate concentration of aromatics, 0.5 mM with 0.5% DMSO, was chosen for the transcriptome induction in *D. squalens*.

### *D. squalens* Showed Specific Transcriptional Response to Lignin-Related Aromatic Compounds

**Figure 2:**
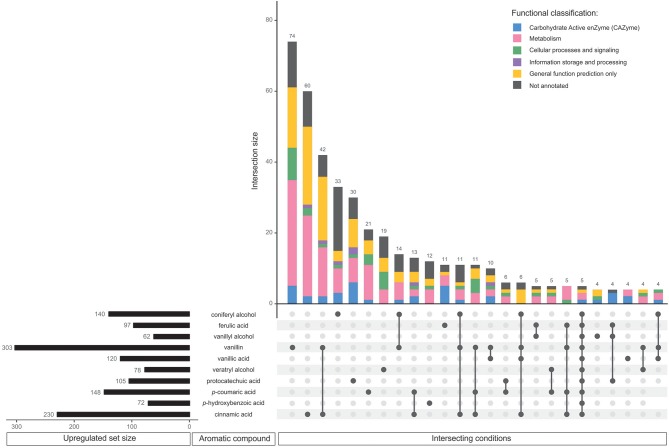
Comparative analysis of all upregulated genes in *D. squalens* grown in the presence of aromatic compounds. The horizontal bars represent the total number of genes identified as upregulated on the individual aromatic compounds using criteria specified in the Materials and Methods. The vertical bars or intersections represent the number of genes that were regulated by one or more aromatic compounds (intersecting conditions). The intersections were organized by size and top 25 sets with the highest number of genes were presented. The genes in each intersection were color-coded according to their predicted functional classification.

**Figure 3:**
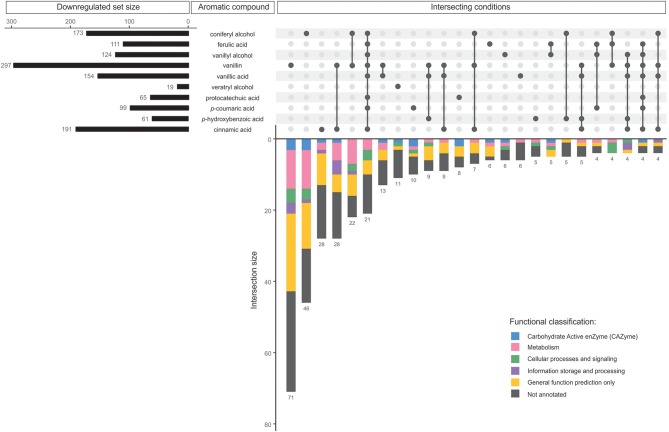
Comparative analysis of all downregulated genes in *D. squalens* grown in the presence of aromatic compounds. The horizontal bars represent the total number of genes identified as downregulated on the individual aromatic compounds. The vertical bars or intersections represent the number of genes that were regulated by one or more aromatic compounds (intersecting conditions). The intersections were organized by size and top 25 sets with the highest number of genes were presented. The genes in each intersection were color-coded according to their functional classification.

Gene expression in *D. squalens* exposed to the monomeric aromatic compounds was analyzed by RNA-seq. Genes with FPKM < 10 in all tested conditions were considered not expressed and excluded from the analysis. Correlation matrix and PCA analysis of the remaining set of 8,733 expressed genes showed high correlation between the biological triplicate cultivations, but also close similarity between different aromatic compounds (correlation coefficient 0.95–1; Supplementary Table 1), suggesting that the most differences are in the subsets of genes. Regulons, i.e., the sets of the regulated genes, were defined as the number of the differentially expressed genes in *D. squalens* cultures supplemented with aromatic compounds when compared to the control conditions using the cutoff values described in Material and Methods. Control cultivations were performed with 0.05% glycerol and 0.5% DMSO without addition of aromatics. It is worth mentioning that the sum of the genes identified in each aromatic regulon was higher than the total number of the regulated genes due to the fact that some genes were co-regulated by two or more aromatic compounds. Expression of all genes identified as up- and downregulated in *D. squalens* in the presence of aromatic compounds can be found in the Supplementary Tables 1, 2. Regulons for each aromatic compound were compared to identify sets of commonly and uniquely affected genes (Figures 2, 3 and Table 1).

**Table 1:** Number of genes differentially expressed by *D. squalens* in response to one or more aromatic compounds (intersecting conditions).

**No. of intersecting conditions**	**1**	**2**	**3**	**4**	**5**	**6**	**7**	**8**	**9**	**All**
Upregulated genes	268 (46.9%)	127 (22.2%)	60 (10.5%)	44 (7.7%)	24 (4.2%)	18 (3.2%)	10 (1.8%)	6 (1.1%)	9 (1.6%)	5 (0.9%)
Downregulated genes	197 (40.9%)	97 (20.1%)	66 (13.7%)	39 (8.1%)	22 (4.6%)	19 (3.9%)	15 (3.1%)	23 (4.8%)	4 (0.8%)	0 (0%)

*Genes that were up- or downregulated in response to unique aromatic compound were considered to be part of specific transcriptional response in D. squalens. Total of 1,053 genes were differentially expressed in response to aromatic compounds, of which 571 were upregulated and 482 downregulated*.

Among the upregulated genes, 268 (46.9%) were affected by a single aromatic compound. Notably lower number of upregulated genes was affected by more than one of the tested aromatic compounds (Table 1). Vanillin upregulated the largest set of genes (303) and also the largest number of uniquely upregulated genes (74; Figure 2). Large number of these genes was functionally associated with metabolism, although genes with only general prediction available were also abundant. Cinnamic acid upregulated the second largest regulon (230 genes) and the second largest unique intersection (60 genes). The third largest group of uniquely upregulated genes (42) responded to both vanillin and cinnamic acid. Similarly, aromatics that upregulated large regulons (coniferyl alcohol, 140 genes; *p*-coumaric acid, 148 genes) are among the conditions with the highest uniquely upregulated intersections (>20 genes). However, protocatechuic acid upregulated more specific genes (30) than *p*-coumaric acid (21), despite much smaller regulon (105). On vanillic acid, which upregulated one of the largest sets of genes (120), only four genes were specifically affected. It is also worth mentioning that out of the 10 largest intersections, seven are upregulated specifically by a single aromatic compound (Figure 2).

In total 197 (40.9%) of all, downregulated genes were affected by a single aromatic compound (Table 1). Accordingly with the upregulated genes, much lower number of the genes were affected by two or more aromatic compounds. None of the genes was downregulated in response to all 10 aromatic compounds. Similarly to the upregulated regulons, vanillin downregulated the largest set of genes (297) including 71 specifically affected genes (Figure 3). In addition, the downregulated genes included much lower number of putative metabolic genes and large set of genes with poor or not existing annotation. Remarkably, out of 14 intersections containing two or more conditions, vanillin was involved in co-regulation of 11 intersections of genes (Figure 3). Coniferyl alcohol and cinnamic acid downregulated 46 and 28 unique genes, respectively, followed by another 28 genes, which were downregulated by both vanillin and cinnamic acid.

### Aromatic Monomers Induce Expression of Genes Relevant for the Utilization of Lignocellulose

#### Gene Ontology Enrichment Analysis

**Figure 4:**
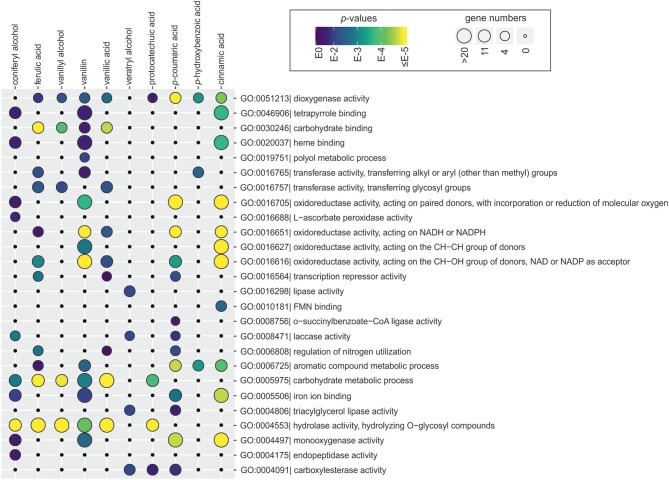
Gene Ontology (GO) terms associated with the function of genes upregulated in the presence of aromatic compounds in *D. squalens*. The size and color of the circles represent the number of genes and statistical significance of enriched GO terms, respectively. The GO terms with redundant biological role were manually removed on the figure. The full list of enriched GO terms can be found in Supplementary Table 3.

To identify cellular processes that are activated and repressed in response to each aromatic compound, all functionally annotated genes were subjected to the Gene Ontology (GO) enrichment analysis. Many GO terms linked to aromatic metabolism (oxidative activities) and lignocellulose utilization (hydrolytic activities, carbohydrate binding and metabolism, and lignin-modifying activities) were over-represented among upregulated genes (Figure 4 and Supplementary Table 3). Several broad GO terms related to oxidative activity were commonly enriched among genes upregulated by tested aromatic compounds. These included e.g., dioxygenase activity (GO:0051213) that was enriched on ferulic acid, vanillyl alcohol, vanillin, vanillic acid, protocatechuic acid, *p*-coumaric acid, *p*-hydroxybenzoic acid and cinnamic acid, and oxidoreductase activity acting on the CH-OH group of donors, NAD or NADP as acceptor (GO:0016616) that was enriched on ferulic acid, vanillin, vanillic acid, *p*-coumaric acid, and cinnamic acid (Figure 4). Nonetheless, differences between aromatics were found at the higher specificity level, for example direct descendants (child terms) of GO:0016616, such as epoxide dehydrogenase activity and phenylcoumaran benzylic ether reductase activity were only enriched on vanillin and cinnamic acid, while 3-oxoacyl-[acyl-carrier-protein] reductase activity was specifically enriched on vanillic acid (Supplementary Table 3).

GO terms related to hydrolytic activity acting on glycosyl bonds (GO:0004553) were commonly enriched among upregulated genes, including mannosidase, fucosidase, and amylase activities on coniferyl alcohol, ferulic acid, vanillyl alcohol, vanillin and vanillic acid, and polygalacturonase activity on ferulic acid and vanillyl alcohol (Figure 4 and Supplementary Table 3). GO terms associated with lignin oxidation, including laccase activity and copper ion binding, were over-represented among genes upregulated by coniferyl alcohol, veratryl alcohol, and *p*-coumaric acid. GO terms linked to other metabolic processes were less abundant, such as lipase activity uniquely enriched on veratryl alcohol and peptidase activity on coniferyl alcohol. Interestingly, two GO terms related to transcriptional regulation, i.e., regulation of nitrogen utilization and transcription repressor activity, were enriched on ferulic acid, vanillic acid, and *p*-coumaric acid.

Among the downregulated genes, notably lower number of enriched GO terms was found. Several GO terms related to structural elements of cell wall (GO:0005199) were overrepresented among genes downregulated by coniferyl alcohol, ferulic acid, vanillyl alcohol, and vanillin (Supplementary Table 3). Vanillin, vanillic acid, and cinnamic acid also downregulated genes enriched in metal ion binding (iron, heme). Additionally, few GO terms related to oxidoreductase activity (e.g., GO:0016491, GO:0016705) with lower enrichment score were identified among genes downregulated in the presence of cinnamic acid, vanillic acid, vanillin, and ferulic acid.

#### Metabolic Genes Important for Conversion of Aromatics

**Figure 5:**
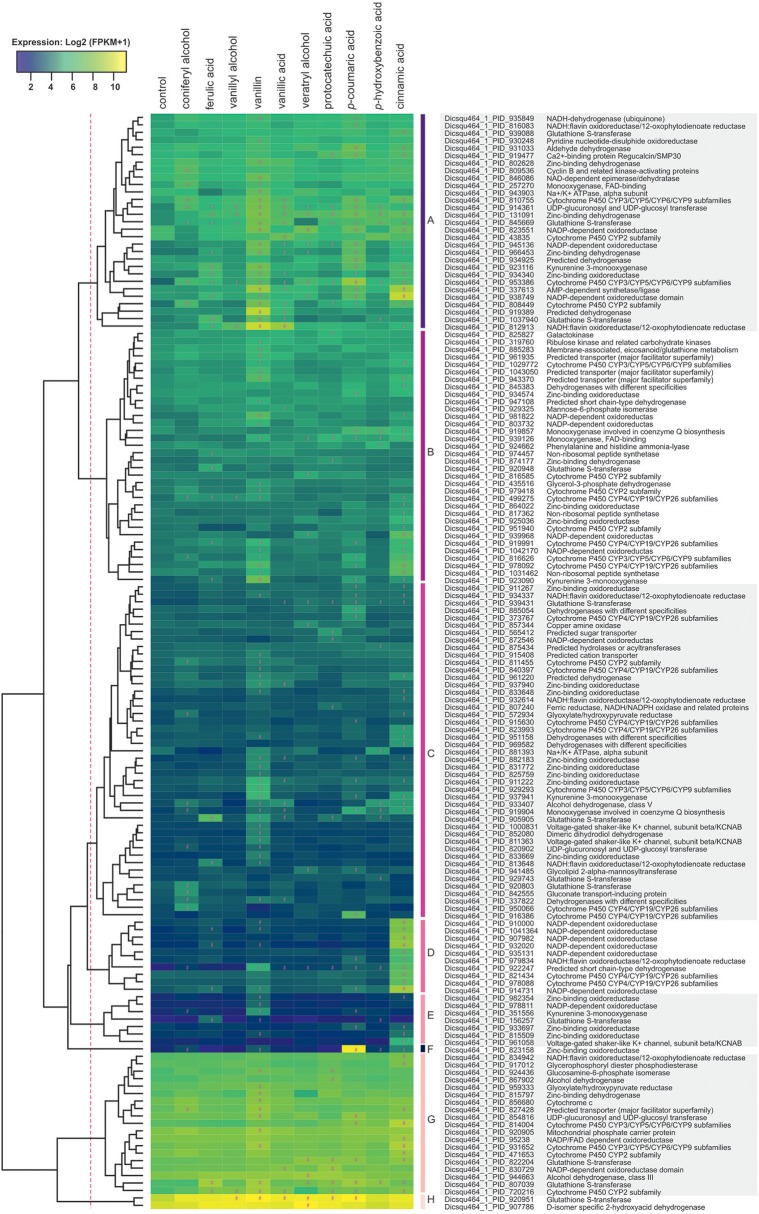
Hierarchical clustering of metabolic genes that were upregulated in the presence of aromatic compounds in *D. squalens*. The color code represents average and log_2_ expression values (FPKM+1) from biological triplicate cultures. Significantly upregulated genes are marked with a hashtag sign (#). The protein ID and (putative) function are shown on the right. Genes that belong to “Amino acid transport and metabolism” (29), “Lipid transport and metabolism” (14), and “Nucleotide transport and metabolism” (2) classes were omitted.

To facilitate the functional analysis, all differentially expressed genes were tentatively assigned to groups using the above-mentioned KOG-based classification system (Supplementary Table 1; columns: assigned functional group and class). The presence of lignocellulose-related aromatic compounds, induced expression of 192 putative metabolic genes (43% of all upregulated genes) in *D. squalens*, including large set of putative cytochrome P450 (cytP450) and aldehyde dehydrogenase encoding genes that may be involved in aromatic metabolism (Supplementary Table 1). Additionally, several differentially expressed genes were identified as best hits in protein sequence homology search using previously characterized bacterial and fungal aromatic enzymes as query (Table 2, Supplementary Table 4). Expression-based hierarchical clustering of 147 metabolic genes that were considered relevant to lignin utilization led to the identification of eight clusters of upregulated genes (Figure 5, Clusters A–H). Upregulated metabolic genes associated with amino acid (29), lipid (14), and nucleotide (2) transport and metabolism (Supplementary Table 1) were omitted when drawing Figure 5, as they are less likely to be related to aromatic metabolism and lignin utilization. Cluster A contains genes with high expression levels (>70 FPKM) only in the presence of aromatic compounds, including four genes encoding putative cytP450 oxidoreductases (Dicsqu464_1_PID_810755, Dicsqu464_1_PID_43835, Dicsqu464_1_PID_953386, Dicsqu464_1_PID_808449) and seven genes encoding other oxidoreductases (Dicsqu464_1_PID_816083, Dicsqu464_1_PID_930248, Dicsqu464_1_PID_802628, Dicsqu464_1_PID_131091, Dicsqu464_1_PID_966453, Dicsqu464_1_PID_934340, Dicsqu464_1_PID_812913). Interestingly, Dicsqu464_1_PID_816083 and Dicsqu464_1_PID_812913 had low sequence homology to salicylyl-CoA 5-hydroxylase SdgC, which is involved in salicylic acid pathway in *Streptomyces* sp. (Ishiyama et al., 2004). Majority of genes in cluster A, 20 out of 29, were co-regulated by more than one aromatic compound. These include two genes (Dicsqu464_1_PID_914361 encoding putative UDP-glucosyl transferase and Dicsqu464_1_PID_953386 encoding cytP450) that were induced by all 10 compounds and another two genes (Dicsqu464_1_PID_131091 encoding putative zinc-binding oxidoreductase and Dicsqu464_1_PID_845669 encoding putative glutathione S-transferase) that were affected by nine aromatics. Vanillin, *p*-coumaric acid, and cinnamic acid had the strongest effect and upregulated 22, 18, and 17 genes from cluster A, respectively. Genes in clusters B, C, and E were moderately expressed in the presence of the studied aromatic compounds. Vanillin and cinnamic acid upregulated the highest number of genes: 19 and 18 in cluster B, 24 and 15 in cluster C, and 5 and 4 in cluster E, respectively. Genes in cluster D were strongly upregulated in the presence of cinnamic acid and contained six putative NADP-dependent oxidoreductases (Dicsqu464_1_PID_910000, Dicsqu464_1_PID_1041364, Dicsqu464_1_PID_907982, Dicsqu464_1_PID_932020, Dicsqu464_1_PID_93513, Dicsqu464_1_PID_914731) and two cytP450 encoding genes (Dicsqu464_1_PID_821434, Dicsqu464_1_PID_978088). Cluster F contains a single gene encoding zinc-binding oxidoreductase that was very strongly upregulated in the presence of *p*-coumaric acid (928-fold change when compared to control). Clusters G and H contain genes, which were highly expressed in the control samples. However, their expression showed significant increase with some of the aromatic compounds. Interestingly, 10 out of 19 genes in cluster G were uniquely upregulated by a single aromatic compound.

**Table 2:** Differentially expressed *D. squalens* genes with low to medium level of sequence homology to previously identified bacterial (Atu1415, BadA, BagX, BclA, BenD, CalA, CalB, CouL, Fcs, HapB, HbaA, PobA, XlnD, SdgC, NahG) and fungal (BphA, CprA, Phhy, PcCYP1f, CYP53A15) aromatic enzymes.

**Protein ID**	**Best hit for (% hit coverage/** **% hit identity)**	**Control**	**Coniferyl alcohol**	**Ferulic acid**	**Vanillyl alcohol**	**Vanillin**	**Vanillic acid**	**Veratryl alcohol**	**Protocatechuic acid**	***p*-coumaric acid**	***p*-hydroxy-benzoic acid**	**Cinnamic acid**
416914	*p*-Hydroxyphenoxy-β-hydroxyacyl-CoA dehydrogenae Atu1415 (31.56%/40.86%), Benzoate 1,2-dioxygenase BenD (39.92%/40.28%)	38.80	35.12	49.66	49.84	32.28	26.44	59.61	35.56	38.69	22.33	77.62
974457	Benzoate-CoA ligase BadA (16.84%/30.46%), Feruloyl-CoA synthase Fcs (15.39%/43.4%), 4-Hydroxybenzoate-CoA ligase HbaA (16.36%/33.73%), Salicylyl-AMP ligase SdgA (15.2%/32.48%)	17.41	15.81	33.66	29.65	27.99	32.58	23.09	20.25	20.64	17.75	20.97
937941	3-Hydroxybenzoate 6-hydroxylase BagX (38.8%/35.43%) and XlnD (38.14%/40.12%), Salicylate hydroxylase NahG (37.25%/40.48%)	6.20	5.64	5.92	3.96	65.26	8.88	6.12	6.44	14.87	5.82	36.33
941829	Benzoate 1,2-dioxygenase BenD (54.98%/44.2%)	5.85	3.47	4.32	5.21	52.44	5.58	6.32	6.18	4.80	5.55	5.44
814004	Benzoate 4-monooxygenase BphA (78.89%/56.57%) and BzuA (82.78%/53.02%), high homology to PcCYP1f and CYP53A15	120.15	105.00	93.75	84.17	172.24	127.83	112.43	172.44	293.26	215.09	672.43
933407	Coniferyl alcohol dehydrogenase CalA (62.97%/32.41%)	6.47	18.94	10.42	3.18	21.72	26.00	3.56	4.29	8.10	32.53	33.80
944663	Coniferyl alcohol dehydrogenase CalA (15.83%/40%)	254.71	293.32	403.89	401.00	395.35	377.30	499.35	476.23	373.26	271.77	252.50
931033	Coniferyl aldehyde dehydrogenase CalB (68.48%/36.65%)	49.05	72.69	60.61	62.57	81.69	55.57	92.79	63.18	175.47	52.98	94.27
95238	Cytochrome P450 reductase CprA (76.89%/50.27%)	255.90	276.98	263.57	257.70	399.19	270.07	241.24	225.57	295.19	271.23	490.25
826556	4-Hydroxyphenylacetate esterase HapB (49.48%/40.14%)	36.04	41.89	52.28	52.36	65.83	54.86	73.38	44.51	59.65	48.04	50.72
485773	4-Hydroxyphenylacetate esterase HapB (42.6%/36.81%)	57.59	94.48	137.58	113.06	259.10	89.72	128.15	71.71	165.76	70.93	228.08
919857	Phenol hydroxylase PhhY (77.48%/50.3%)	28.90	17.98	25.56	24.53	20.76	25.18	28.04	30.73	72.59	73.58	58.87
919904	Phenol hydroxylase PhhY (75.12%/46.49%)	4.20	9.98	5.03	4.14	14.94	10.37	5.67	5.65	46.25	17.25	38.02
351556	*p*-Hydroxybenzoate-*m*-hydroxylase PobA (17.75%/45.07%)	1.92	4.08	2.33	1.64	30.34	2.66	2.80	2.95	4.98	3.75	2.53
834942	Salicylyl-CoA 5-hydroxylase SdgC (57.87%/40.59%)	100.15	104.67	110.27	107.04	139.98	112.45	110.58	157.76	140.45	113.81	248.15
813648	Salicylyl-CoA 5-hydroxylase SdgC (26.33%/52.53%)	4.86	5.62	23.96	4.09	15.07	5.07	5.41	4.75	6.85	5.16	3.91
816083	Salicylyl-CoA 5-hydroxylase SdgC (26.03%/48.51%)	35.98	63.37	47.78	51.56	63.56	40.63	60.82	56.43	82.57	44.58	62.69
812913	Salicylyl-CoA 5-hydroxylase SdgC (36.73%/39.42%)	22.97	22.65	60.33	109.77	1071.38	473.08	47.31	58.65	50.88	36.22	57.09
923090	3-Hydroxybenzoate 6-hydroxylase XlnD (39.16%/37.85%)	8.02	11.03	17.45	10.85	175.42	11.63	9.47	11.45	36.57	12.33	29.14

*For comprehensive review of characterized bacterial and fungal aromatic metabolic enzymes see Lubbers et al. (2019). Genes with fold change > 2 and adjusted p-value < 0.01 in the presence of aromatics when compared to the control conditions without aromatics were highlighted in gray*.

#### Genes Encoding Plant Cell Wall Modifying Enzymes

**Figure 6:**
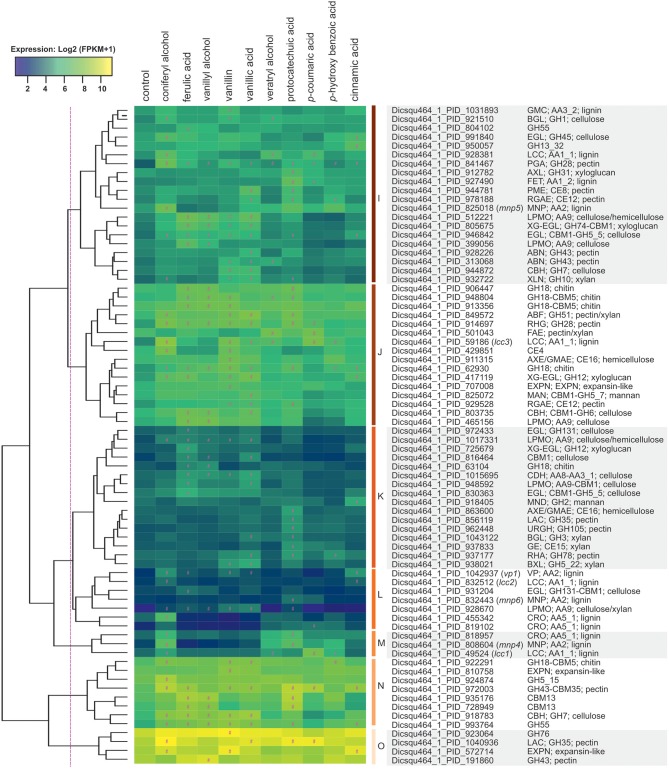
Hierarchical clustering of CAZy genes that were upregulated in the presence of aromatic compounds in *D. squalens*. The color code represents average and log_2_ expression values (FPKM+1) from biological triplicate cultures. Significantly upregulated genes are marked with a hashtag sign (#). The protein ID and (putative) function are shown on the right.

In total 74 (13%) of all upregulated genes encoded putative Carbohydrate-Active enZymes (CAZymes), including 53 and 13 that were related to plant polysaccharide and lignin degradation, respectively. Vanillic acid, vanillin and ferulic acid upregulated the largest sets of polysaccharide-related genes (> 22 each), while veratryl alcohol, *p*-coumaric acid and *p*-hydroxybenzoic acid induced the smallest sets (< five genes each). Majority of induced genes related to lignin-degradation responded to coniferyl alcohol (nine out of 13), while vanillyl alcohol did not induce any lignin-degradation related genes. Induced CAZyme encoding genes clustered based on their expression into seven groups (Figure 6). Cluster I include four putative lignin-, eight (hemi)cellulose-, and five pectin-related CAZyme encoding genes. One laccase encoding gene (Dicsqu464_1_PID_928381) was induced 3.5-fold by coniferyl alcohol, 2.8-fold by veratryl alcohol, and 2.5-fold by *p*-coumaric acid, while one manganese peroxidase (MnP) encoding gene (Dicsqu464_1_PID_825018) was 4.2-fold upregulated only in the presence of the monolignol coniferyl alcohol. Cluster K includes lower expressed genes and genes putatively encoding enzymes involved in (hemi)cellulose and pectin degradation, whereas majority of genes in clusters L and M are predicted to be involved in lignin-degradation (eight out of ten). Coniferyl alcohol induced the highest number of genes (seven out of ten) in clusters L and M when compared to other aromatic compounds. Despite low expression in control conditions, cluster L contains interesting expression patterns including one versatile peroxidase (VP) encoding gene (Dicsqu464_1_PID_1042937), which was 6.7-fold up-regulated by coniferyl alcohol, 7.6-fold by cinnamic acid, and 4.0-fold by vanillin. Cluster M contains one *mnp* gene (Dicsqu464_1_PID_808604) strongly up-regulated (>14-fold increase) by coniferyl alcohol and protocatechuic acid, and one laccase gene (Dicsqu464_1_PID_49524) strongly upregulated (12.4-fold increase) by *p*-coumaric acid among others.

Clusters J and N included genes that were highly expressed (>70 FPKM) only in the presence of aromatic compounds. Vanillin induced the highest number of genes (11) in cluster J, followed by ferulic and vanillic acid (8 genes both). One laccase encoding gene (Dicsqu464_1_PID_59186) was strongly upregulated in the presence of coniferyl alcohol showing 10.5-fold increase, while vanillin caused 6.3-fold and *p*-coumaric acid 6.9-fold induction, and 3.3-, 3.5-, and 3.3-fold increase was caused by vanillic acid, veratryl alcohol, and *p*-hydroxybenzoic acid, respectively. The genes encoding putative pectin-acting enzymes in cluster J were generally upregulated in the presence of vanillin, vanillic acid and protocatechuic acid, with exception of a feruloyl esterase encoding gene (Dicsqu464_1_PID_501043) that was induced only by veratryl alcohol and *p*-coumaric acid. Cluster O contain genes with the highest expression among all CAZymes. These genes encoded mainly putative glycoside hydrolase (GH) enzymes related to pectin degradation.

#### Other Differentially Expressed Genes

Beside metabolic enzymes and CAZymes encoding genes, aromatic compounds induced expression of several other genes that could be relevant for lignocellulose degradation in *D. squalens*. Upregulated genes included 17 putative transporters (three ABC and 14 Major Facilitator Superfamily transporters), 14 genes involved in defense mechanisms/metabolism (mainly NAD-dependent epimerase/dehydratases), and four putative hydrophobin encoding genes tentatively assigned to “cellular processes and signaling” group (Supplementary Table 1). Additionally, hierarchical clustering analysis of genes with only partial annotation or not included in the traditional KOG classification (here tentatively assigned to a group with “general function prediction only”), showed interesting expression patterns (Supplementary Figure 2). Genes from cluster P and R were especially highly expressed in the presence of aromatic compounds, and at least 20 had putative metabolic function related to oxidoreductase activity and therefore could be related to aromatic metabolism. Interestingly, one putative intradiol ring-cleaving dioxygenase encoding gene, Dicsqu464_1_PID_972012, clustered together with the highest expressed genes in this group and was additionally upregulated on vanillin, *p*-coumaric acid, *p*-hydroxybenzoic acid, and cinnamic acid (Supplementary Figure 2, Cluster R). There are two types of aromatic ring-cleaving enzymes, intradiol dioxygenases, which cleave aromatic ring between the OH groups and extradiol dioxygenases, which cut next to the OH group (Lubbers et al., 2019). Besides Dicsqu464_1_PID_972012, *D. squalens* expressed second putative intradiol dioxygenase, Dicsqu464_1_PID_819116, and one putative extradiol dioxygenase, Dicsqu464_1_PID_963638. While both genes were not highly expressed, their expression was upregulated by nearly all tested aromatic compounds (nine out of ten, Supplementary Table 1). Another important group of genes included three upregulated putative transcription factor encoding genes (“information storage and processing”), while additional three putative transcription factor candidates were found in the group of genes with general function prediction only (Supplementary Figure 2). Moreover, large part of the transcriptome upregulated in *D. squalens* in the presence of aromatic compounds was very poorly annotated, including many gene models without any annotation available (Figures 2, 3 and Supplementary Table 1). Expression of these genes was analyzed using hierarchical clustering and several highly expressed clusters strongly affected by the aromatic compounds were identified (Supplementary Figure 3).

## Discussion

Aromatic components present in lignocellulose, especially in the aromatic lignin polymer hold a great potential for production of renewable high-value products, e.g., multifunctional aromatic compounds that could be used as alternative to fossil fuel-derived chemicals or their precursors (Li et al., 2015; Feghali et al., 2018). While many microbes can degrade lignocellulose, white-rot basidiomycete fungi are the only organisms in nature that break down all the polymeric components of lignocellulose, including recalcitrant lignin (Rytioja et al., 2014; Manavalan et al., 2015; Sista Kameshwar and Qin, 2018). Moreover, these fungi have developed a complex metabolic system for conversion of lignocellulose-related aromatic compounds (Mäkelä et al., 2015), which makes them interesting candidates to study especially with relation to lignin valorization.

In this study, we used genome-wide transcriptomic analysis to provide the first systematic insight into conversion of various lignocellulose-related aromatic compounds by the well-studied white-rot fungus *D. squalens* (Dong et al., 2014; Rytioja et al., 2015, 2017; Casado López et al., 2017; Daly et al., 2018). While previous works have applied high-throughput RNA sequencing (RNA-seq) to identify upregulated transcripts from *D. squalens* cultivated on various lignocellulosic substrates (Rytioja et al., 2017; Daly et al., 2018) and lignocellulose-derived monomeric sugars (Casado López et al., 2018), providing novel insights into plant biomass degrading machinery and transcriptional response in this fungus, its molecular response to lignocellulose-derived aromatic compounds has not been studied so far. However, the potential of *D. squalens* for diverse aromatic conversions has been indicated (Daly et al., 2018; Marinović et al., 2018), but more detailed studies are needed to advance our understanding of these processes before assessing their potential in biotechnological applications.

We selected 10 aromatic compounds, which were used as inducing compounds in the *D. squalens* cultures, possibly originating from fungal lignin degradation process (cinnamic acid, coniferyl alcohol) and/or aromatic metabolism (ferulic acid, vanillin, vanillyl alcohol, vanillic acid, veratryl alcohol, protocatechuic acid, *p*-coumaric acid, *p*-hydroxybenzoic acid) (Bugg et al., 2011; Mäkelä et al., 2015). Although, cinnamic acid is an amino acid derived precursor for synthesis of monolignols (Maeda and Dudareva, 2012), it has previously been identified as one of the products of technical Kraft lignin degradation by the bacteria *Bacillus* sp. and *Cupriavidus basilensis* B-8 (Raj et al., 2007; Shi et al., 2013). Some of the tested aromatics, i.e., coniferyl alcohol, ferulic acid and *p*-coumaric acid, also represent naturally occurring *p*-hydroxyphenyl and guaiacyl units of lignin.

Generally, low molecular weight phenolic compounds are toxic to fungi at relatively low concentrations, but differences between compounds and fungal species have been reported (Buswell and Eriksson, 1994; Guiraud et al., 1995; Neves et al., 2005). None of the 10 aromatics tested in this study inhibited growth of *D. squalens* at 0.2 mM concentration. However, higher concentrations (0.5 and 1 mM) of cinnamic acid and vanillin had clearly higher inhibitory effect than the other tested compounds (Figure 1). This is in agreement with a previous study, which showed that 1 mM vanillin inhibited growth by 5–39% in eight white-rot fungi, while 1 mM cinnamic acid abolished growth of six out of the eight species (Buswell and Eriksson, 1994). In the yeast *Saccharomyces cerevisiae*, degree of toxicity was shown to be related to the functional groups on the aromatic compounds, with aldehydes being the most toxic (Ando et al., 1986; Klinke et al., 2003). However, other studies have indicated that additional functional groups also affect the overall toxicity (Adeboye et al., 2014). In *D. squalens*, the particularly strong effect of vanillin (phenolic aldehyde) and cinnamic acid (containing alkene and carboxylic acid groups) when compared to the other aromatic compounds (phenolic acids and alcohols) could be explained by their chemical character and/or presence of functional groups. Nevertheless, higher concentration (1 mM) of all aromatic compounds, except for *p*-hydroxybenzoic acid, inhibited the radial growth of *D. squalens* when compared to lower concentrations of aromatics (Figure 1). Although the amount of these aromatic compounds under natural wood degrading conditions are not known, this suggests that *D. squalens* has not been adapted to encounter very high concentrations of the aromatic monomers in its biotope. The fungus together with other microbes present in the natural community may efficiently convert these compounds already at lower concentrations, thus preventing accumulation of toxic levels of aromatics. Interestingly, brown discoloration of the media was observed in the presence of coniferyl alcohol, ferulic acid, vanillyl alcohol, vanillin, vanillic acid, and protocatechuic acid, suggesting a possible production of an unknown dark-colored metabolite in those cultures (Figure 1). We hypothesize that this phenomenon could be related to the formation of quinones (e.g., methoxyhydroquinone, hydroxyquinol, benzoquinone). Polymerized quinones, which are produced from aromatic compounds naturally present in some fruits have been linked to brown discoloration known as fruit browning (Schieber, 2018). Conversion of vanillic acid, and thus also vanillin, vanillyl alcohol, ferulic acid, and coniferyl alcohol, to methoxyhydroquinone by decarboxylating vanillate hydroxylase and further to hydroxyquinol has been reported in several white-rot fungi, including *Phanerochaete chrysosporium* (Yajima et al., 1979) and its anamorph *Sporotrichum pulverulentum* (Buswell et al., 1981), suggesting it is a common pathway among wood-degrading basidiomycetes. However, conversion of protocatechuic acid to hydroxyquinone have been so far only reported in the yeast *Candida parapsilosis* (Eppink et al., 1997; Holesova et al., 2011).

The molecular responses of *D. squalens* to lignin-related aromatic compounds were analyzed at transcriptome level after induction with 0.5 mM concentration of the selected aromatics. While previous reports investigated the inducing effect of various aromatic compounds on expression of several laccase-encoding genes (Terrón et al., 2004; Piscitelli et al., 2011; Yang et al., 2013, 2016; Moiseenko et al., 2018) in basidiomycete fungi, this is, to the best of our knowledge, the first study that addresses the genome-wide transcriptional response to aromatics and therefore provides a new level of width and depth. The Pearson correlation matrix of all expressed genes (8,733 genes with FPKM >10 in at least one condition) demonstrated high correlation between the biological triplicates, but also between several different aromatics (Supplementary Figure 1), suggesting that the genome-wide response toward different aromatic compounds in *D. squalens* is relatively similar. This may be due to the chemical character of the tested compounds that are all small molecular weight phenylpropane derivatives. However, the two-dimensional PCA highlighted differences between the cultivations induced with the aromatics and the control without aromatics (Supplementary Figure 1). Identification of differentially expressed genes showed that the main differences in mRNA levels were restricted to subsets of genes, and ~6.5 and 5.5% of all expressed genes were identified as up- and downregulated, respectively (Table 1). Moreover, comparative analyses demonstrated that nearly half of these genes were specifically triggered in response to a single aromatic compound (46.9 and 40.9% of up- and downregulated genes, respectively; Table 1) in *D. squalens*. Expression of much lower number of genes was affected by two or more aromatics, and expression of <1% of genes was affected by all the 10 tested compounds. This was further visualized by plotting the top 25 intersections with the highest number of differentially expressed genes and analysing their respective regulating conditions (Figures 2, 3). Respectively, seven and five out of 10 of the biggest intersections were up- and downregulated by a single aromatic compound, indicating very specific transcriptional response of *D. squalens* to lignocellulose-related aromatics. Although, single aromatic compounds affected the highest intersections of both up- and downregulated genes, the specificity seems to be stronger among upregulated genes.

Specifically regulated genes in *D. squalens* were tentatively assigned to six main KOG-related groups based on their functional annotation (Figures 2, 3), which highlighted functional distribution among intersecting conditions. Unfortunately, it also pointed out that KOG categorization is rather outdated in this species and the information between KOG and other available databases has not been integrated. While *D. squalens* is certainly not the poorest annotated basidiomycete fungal genome (Casado López et al., 2019), comparison of the available annotations (KOG, InterPro, KEGG, CAZy) revealed large amount of gaps and a need for careful manual curation and functional data integration, which, however, was out of the scope of this study. It should be noted that various functional categorisations may generate minor mistakes, especially when based on partial gene annotation. We focused our analyses on upregulated genes, as these could be directly involved in aromatic metabolism and thus have potential from the biotechnological point of view. Among annotated genes, a large subset was functionally assigned to metabolism (Figure 2) and many enriched GO terms related to oxidative and hydrolytic activities were detected (Figure 4 and Supplementary Table 3), indicating that at least part of the regulon induced in the presence of aromatic compounds in *D. squalens* is involved in aromatic metabolism and lignocellulose utilization. Additionally, genes upregulated in the presence of aromatics were enriched in GO terms related to lipase and endopeptidase activity (Figure 4). Upregulation of genes linked to lipid and amino acid metabolism (Supplementary Table 1) could indicate that the fungus is dealing with an increased lipid and protein turnover e.g., due to aromatics-inflicted injuries on cellular membranes (Fitzgerald et al., 2004; Gu et al., 2015; Wu et al., 2016; Wang et al., 2017).

Fungal aromatic metabolism is not well-understood yet. However, a schema of aromatic metabolism, based on combined information from many bacterial and fungal species, have been proposed including various known conversions of aromatic compounds (Mäkelä et al., 2015; Lubbers et al., 2019). While many enzymes involved in aromatic metabolism have been characterized in bacteria, only a few of these are known in fungi. A large sequence homology search in *D. squalens* CBS464.89 genome sequence using known bacterial and fungal aromatic enzymes as query identified 145 putative proteins. However, due to low or medium homology, these should not be directly treated as orthologs. Additionally, some *D. squalens* genes had sequence homology to several characterized proteins. Nonetheless, 33 of the corresponding genes were upregulated in the presence of tested aromatic compounds (Table 2), making those interesting candidates for further studies. Dicsqu464_1_PID_814004 was identified with high homology to ascomycete fungal cytP450 monooxygenases (PcCYT1f in *Penicillium chrysogenum* and CYP53A15 in *Cochliobolus lunatus*) and benzoate 4-monooxygenases (BphA in *Aspergillus niger* and BzuA in *Aspergillus nidulands*) involved in benzoic acid pathway e.g., hydroxylation of benzoic acid to *p*-hydroxybenzoic acid (van Gorcom et al., 1990; Fraser et al., 2002; Matsuzaki and Wariishi, 2005; Lah et al., 2011). Interestingly, Dicsqu464_1_PID_814004 was upregulated by 6.1-, 2.5-, and 1.9-fold in the presence of cinnamic acid, *p*-coumaric acid, and *p*-hydroxybenzoic acid, respectively (Table 2). According to the aromatic metabolism schema, cinnamic acid can converted to benzoic acid in three steps while *p*-coumaric acid cam be converted to *p*-hydroxybenzoic acid in four steps, which suggest that upregulation of Dicsqu464_1_PID_814004 could indeed be related to benzoic acid metabolism in *D. squalens*. Another cytP450 encoding gene, Dicsqu464_1_PID_95238, which was identified by sequence homology to CprA involved in benzoic acid pathway in *A. niger*, was 2.1-fold upregulated on cinnamic acid. Two genes, Dicsqu464_1_PID_919857 and Dicsqu464_1_PID_919904, with homology to phenol hydroxylase Phhy that catalyzes conversion of phenol to catechol in yeast *Trichosporon cutaneum*, were upregulated on *p*-coumaric acid, *p*-hydroxybenzoic acid and cinnamic acid suggesting a possible link to (*p*-hydroxy) benzoic pathways. Production of phenol by decarboxylation of *p*-hydroxybenzoic acid have been observed in bacteria but not yet in fungi or yeast (Lubbers et al., 2019). While the sequence homology was certainly higher when comparing *D. squalens* with enzymes characterized in ascomycete fungi, such as Aspergilli, it should be remembered that basidiomycete and ascomycete fungi differ in their abilities to degrade lignin and thus could have very different aromatic metabolism. For example, the white-rot fungus *Lentinula edodes* has been described to hydroxylate ferulic acid to 2-hydroxyferulic acid and then to 2,3,4-trihydroxycinnamic acid (Crestini and Sermanni, 1994). This is one example of many basidiomycete-specific metabolic pathways that have not been observed in ascomycetes.

Out of all differentially expressed genes, vanillin and cinnamic acid upregulated the highest number of genes as well as the highest specifically affected intersections (Figure 2), including the largest subset of metabolic genes enriched in several GO terms related to oxidoreductase activity acting on the CH-CH and CH-OH group of donors with NAD or NADP as an acceptor (Figure 4). These genes, annotated as e.g., putative NADP-dependent oxidoreductases or short-chain dehydrogenase/reductases (SDRs), catalyze electron transfer and are involved in extremely vast range of reactions (Sellés Vidal et al., 2018). Accordingly, GO analysis showed that the genes upregulated by both vanillin and cinnamic acid have diverse activities, such as epoxide dehydrogenase and mevaldate reductase, and thus could be involved in several processes (Supplementary Table 3). Other highly enriched GO terms, oxidoreductase activity acting on paired donors with incorporation or reduction of molecular oxygen and monooxidase activities, included genes annotated as e.g., FAD-binding monooxygenases and cytP450 monooxygenases. CytP450 monooxygenases together with gluthatione S-transferases (GSTs) are especially interesting since their encoding genes are very abundant in the genomes of the white-rot fungi and these enzymes are considered to be involved in conversion of toxic aromatic compounds released from wood degradation (Morel et al., 2013; An et al., 2019) and thus have potential for biotechnological applications (Girvan and Munro, 2016). Interestingly, 22 cytP450 and 17 GST encoding genes were induced in the presence of tested aromatic compounds (Figure 5, Supplementary Table 1 and Supplementary Figure 2). Vanillin upregulated the highest number of GSTs (11) on average by 6-fold change, while cinnamic acid upregulated the highest number of cytP450s (15) on average by 5.8-fold. Three of the induced cytP450 encoding genes (Dicsqu464_1_PID_810755, Dicsqu464_1_PID_808449 and Dicsqu464_1_PID_471653) were previously shown to be upregulated in *D. squalens* when grown on birch and spruce wood (Daly et al., 2018) Additionally, vanillin and cinnamic acid were shown to have the most inhibitory effect on *D. squalens* colony growth diameter (Figure 1), thus suggesting that upregulated genes could catalyze conversion of these specific compounds into less toxic ones.

White-rot fungi secrete an array of CAZymes to break large lignocellulose polymers to smaller units that can be taken into the fungal cells, where they are metabolized as sources of carbon and energy or, in the case of aromatics, converted to less toxic compounds. Several CAZyme encoding genes involved in extracellular plant biomass degradation were upregulated in *D. squalens* in response to aromatic compounds (Figures 2, 6). Vanillic acid, vanillin, and ferulic acid upregulated the largest sets of polysaccharide-related genes, including those encoding cellulose and hemicellulose-active enzymes, while veratryl alcohol, *p*-coumaric acid, and *p*-hydroxybenzoic acid induced the smallest sets. Interestingly, ferulic acid induced 22 genes involved in polysaccharide degradation. However, none of these genes encode putative enzymes that cleave the bonds between hemicellulose and lignin. Nonetheless, upregulation of hydrolytic activities by ferulic acid could be linked to the presence of this compound in plant cell wall polysaccharides. All aromatic compounds induced expression of some pectinolytic genes (Figure 6). Pectin, which is present in high concentrations in the bordered pits in plant cell walls, has been suggested to be degraded first in order of the fungus to be able to enter in the plant cell. Previous transcriptome analysis from *D. squalens* mono- and disaccharide cultures demonstrated that cellobiose and L-rhamnose trigger expression of enzymes involved in polysaccharide degradation in *D. squalens* (Casado López et al., 2018), while induction of ligninolytic genes in *D. squalens* has been shown to be specifically triggered in the presence of lignin-rich substrates (Rytioja et al., 2017). However, our study indicated that lignin-degrading enzymatic machinery of *D. squalens* was only partially induced in the presence of the monomeric aromatic compounds. In total 13 lignin-degradation related enzymes encoding genes were upregulated in *D. squalens* exposed to aromatic monomers, majority of which (nine) were induced by coniferyl alcohol alone (two out of nine) or in combination with other aromatics (seven out of nine). Partial induction of laccase encoding genes by aromatic compounds was also shown in other white-rot fungus, *Trametes hirsuta* (Moiseenko et al., 2018). This suggests that lignin-degrading enzymes in the white-rot fungi may not be similarly induced as the polysaccharide degrading ones, i.e., by the monomeric building blocks of the lignin polymer. However, differences between species are also expected. For example, two laccase encoding genes, *lcc3* and *lcc1*, were highly expressed (>70 FPKM) in *D. squalens* grown with *p*-coumaric acid when compared to the control conditions, while in *T. hirsuta* the expression of homologous genes was strongly repressed by *p*-coumaric acid (Moiseenko et al., 2018).

In summary, *D. squalens* showed a prominent ability for conversion of aromatic compounds as indicated by the number of upregulated genes under the studied conditions, indicating a potential of this fungus for bio-based valorization of lignocellulosic biomass. We showed that regulons induced in the presence of aromatic compounds differ with respect to both the number and function of the genes. For example, cinnamic acid, vanillin, and *p*-coumaric acid upregulated the most diverse array of GO terms associated with oxidoreductase activities, while coniferyl alcohol, ferulic acid, and vanillyl alcohol upregulated the highest number of GO terms associated with lignocellulolytic activities. Additionally, we showed that transcriptional response of *D. squalens* to aromatics is largely compound-specific and indicated the set of highly induced oxidative enzymes that are potentially involved in aromatic metabolism.

## Data Availability Statement

The datasets generated for this study can be found in the NCBI with individual sample Accession Numbers from PRJNA500193 to PRJNA500234.

## Author Contributions

JK performed the experiments, analyzed the data, and wrote the paper. MPe contributed to the bioinformatics analysis and data visualization. MPa, AL, VN, VS, MW, and IG performed the RNA sequencing and analysis. MM conceived the study and revised the paper.

### Conflict of Interest

The authors declare that the research was conducted in the absence of any commercial or financial relationships that could be construed as a potential conflict of interest.
