# Supplementary figures and images for: The White-Rot Basidiomycete Dichomitus squalens Shows Highly Specific Transcriptional Response to Lignocellulose-Related Aromatic Compounds
Source: Front Bioeng Biotechnol. 2019 Sep 20;7:229. doi: 10.3389/fbioe.2019.00229 (PMC6763618; doi:10.3389/fbioe.2019.00229)

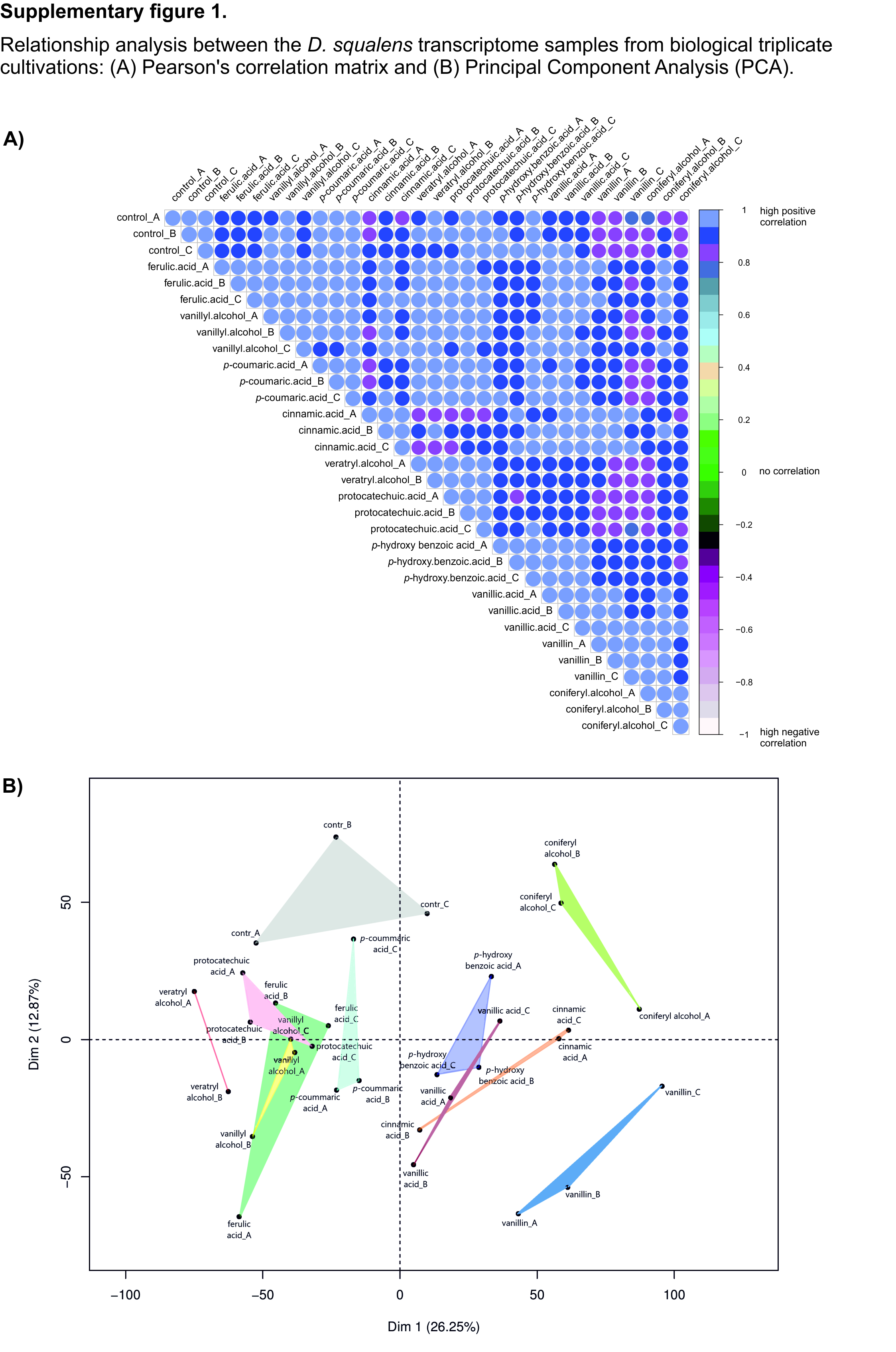

Supplement: Supplementary file 5 [file Image_1.JPEG]

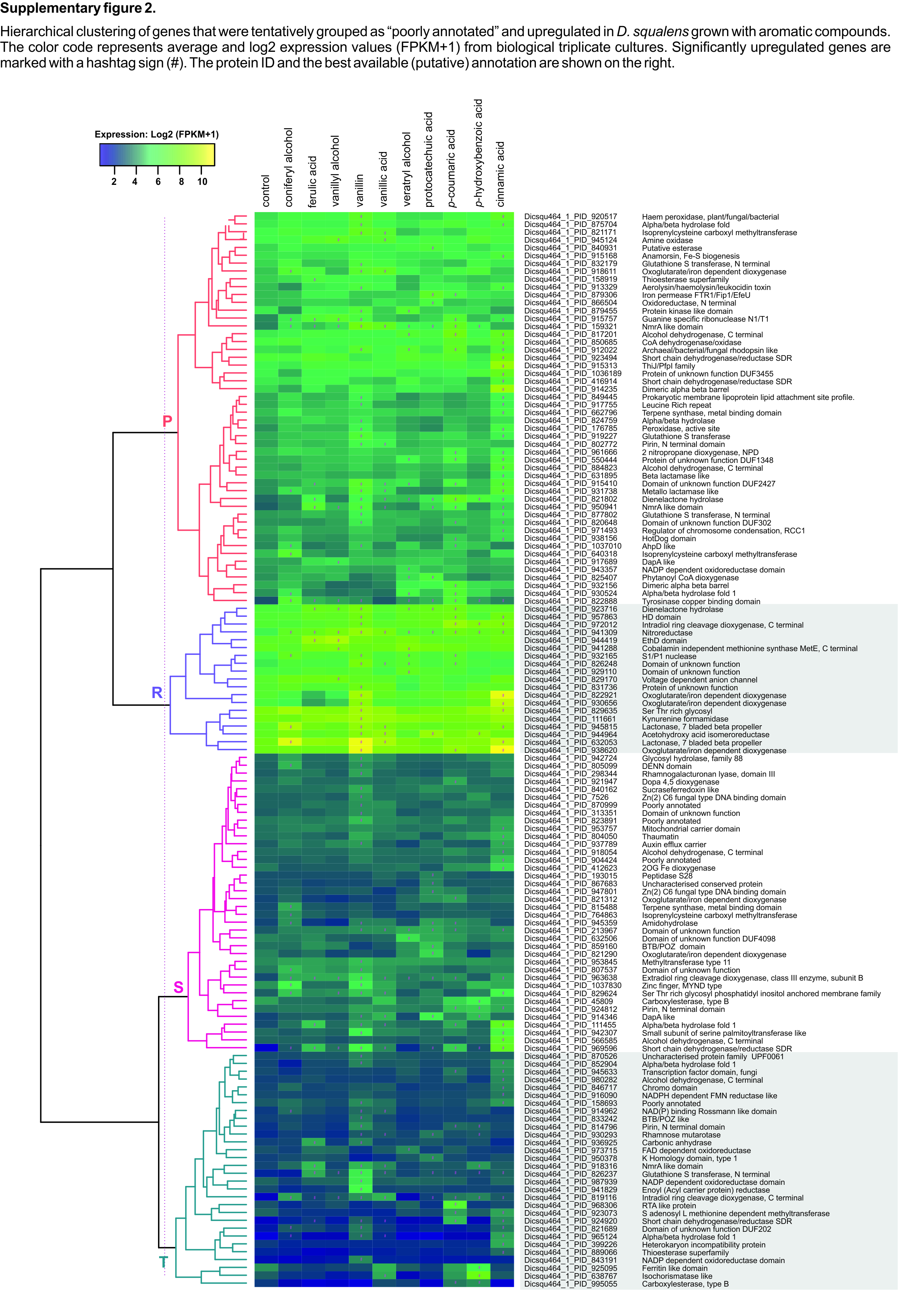

Supplement: Supplementary file 6 [file Image_2.JPEG]

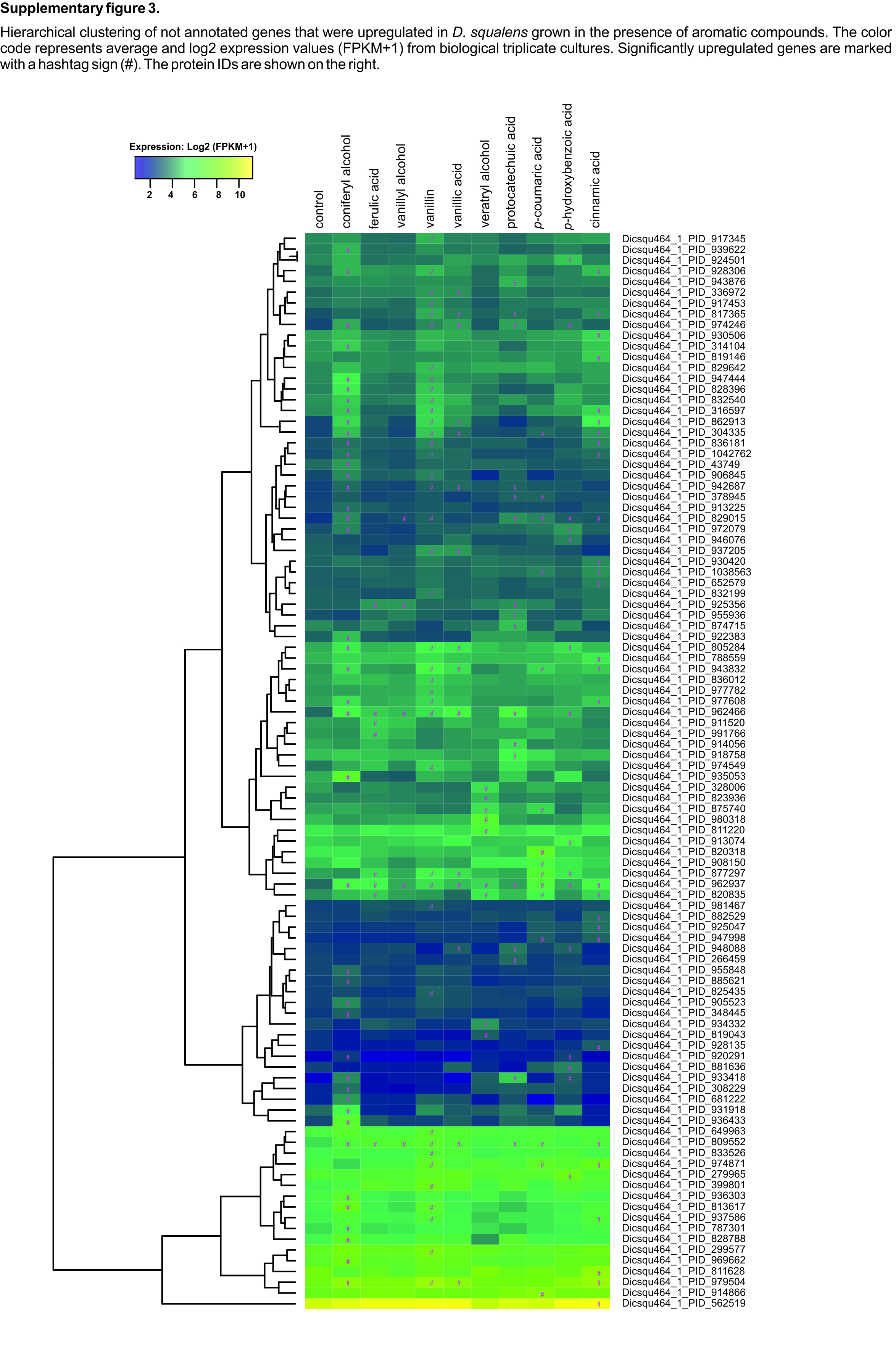

Supplement: Supplementary file 7 [file Image_3.JPEG]
